# The Association Between Community Dialogue and Loneliness in Rural Japanese Communities: A Cross-Sectional Study

**DOI:** 10.7759/cureus.57744

**Published:** 2024-04-06

**Authors:** Ryuichi Ohta, Toshihiro Yakabe, Hiroshi Adachi, Chiaki Sano

**Affiliations:** 1 Community Care, Unnan City Hospital, Unnan, JPN; 2 Family Medicine, Unnan City Hospital, Unnan, JPN; 3 Community Medicine Management, Faculty of Medicine, Shimane University, Izumo, JPN

**Keywords:** family medicine, general medicine, health promotion, cross-sectional studies, loneliness, community networks, social isolation, rural health

## Abstract

Introduction

This investigation explores the influence of community dialogue on loneliness within rural Japanese communities amidst the backdrop of globalization, urbanization, and technological shifts. Highlighting the significance of both informal and formal community dialogues, the study aims to bridge the gap in empirical evidence regarding the role of these interactions in enhancing social cohesion and mitigating loneliness, particularly in rural areas facing demographic changes and privacy concerns.

Method

A cross-sectional study was performed in Unnan City, Japan, targeting individuals over 40 who regularly visited a local rural hospital. The study employed the Japanese version of the University of California, Los Angeles (UCLA) Loneliness Scale and questionnaires regarding the frequency of community dialogue, alongside examining participants' health and demographic details from hospital records. Analysis methods included t-tests, Mann-Whitney U tests, and multivariate logistic regression to examine the association between community dialogue frequency and loneliness.

Results

Among the 647 respondents, the participants’ mean age was 71.26 years, with a male rate of 46.3%. The multivariate logistic regression analysis revealed that higher frequencies of community dialogue significantly reduce the odds of experiencing loneliness. Specifically, compared to individuals with the least frequency of community dialogue, those with more frequent and most frequent dialogues were significantly more likely to report higher loneliness levels, with odds ratios of 2.62 (95% CI: 1.60-4.29, p<0.01) and 4.11 (95% CI: 2.47-6.85, p<0.01), respectively. Additionally, an increase in BMI was inversely related to loneliness (OR: 0.95, 95% CI: 0.91-0.99, p=0.023), and individuals with a higher comorbidity index (CCI≥5) showed a decreased likelihood of reporting higher loneliness (OR: 0.64, 95% CI: 0.43-0.96, p=0.031).

Conclusion

This study shows compelling evidence that more frequent community interactions are inversely associated with feelings of loneliness. These findings suggest that initiatives to increase community dialogue need a nuanced approach to mental health and social cohesion in rural settings. The research further reveals an intriguing relationship between body mass index, the severity of comorbidities, and loneliness, offering insights into the complex interplay between physical health and social well-being. The importance of this study lies in its potential to inform policies and programs designed to foster social connections respecting rural contexts, thereby addressing the challenge of loneliness in rural communities.

## Introduction

In the modern era, the fabric of community cohesion is increasingly tested by globalization, urbanization, and technological advancement [1]. Among these dynamics, community dialogue has emerged as a pivotal element in sustaining the vitality and health of communities [2]. Community dialogue, encompassing informal interactions among friends and formal discussions within community centers, fosters mutual understanding and a culture of support [3,4]. This exchange of ideas and experiences is not merely a social nicety [3,4]. Still, a vital process that promotes a sense of belonging mitigates social isolation and directly impacts the well-being of community members [5]. The significance of such dialogue becomes even more pronounced in rural communities, where demographic shifts and social privacy concerns erode traditional forms of communal interaction [6].

This research focuses on the critical role of community dialogue in sustaining rural communities' health and social cohesion. It posits that community dialogue, whether in informal gatherings or structured forums, is instrumental in building effective relationships among community members [3]. Community dialogue can alleviate social isolation and its attendant health risks by fostering mutual understanding and supporting a culture of mutual assistance. However, the frequency and contents of dialogue vary and may affect their perceptions of lives in their communities [6]. This is especially pertinent in rural areas, where dwindling populations and increased privacy concerns have led to declining communal interactions, exacerbating feelings of loneliness and its harmful health consequences [7].

The gradual erosion of community dialogue in rural areas, driven by demographic changes and heightened social privacy concerns, presents a significant challenge to community sustainability [8]. Loneliness, as a direct consequence of this decline in communal interaction, is not just a personal affliction but a public health issue with far-reaching implications for morbidity and mortality [9]. Despite the intuitive link between community dialogue and social well-being, more empirical evidence needs to delineate the relationship between the presence and quality of community dialogue and the levels of loneliness within rural communities [10,11]. This gap in research underscores the need for a thorough investigation into how community dialogue can be effectively leveraged to combat loneliness and foster a healthier, more cohesive community environment.

The central question guiding this research is as follows: What is the association between community dialogue and the degree of loneliness in rural Japanese communities? This study aims to elucidate this relationship, providing empirical evidence that can inform community-based strategies to enhance dialogue and, by extension, reduce loneliness. By clarifying the extent to which community dialogue influences feelings of loneliness, this research aims to galvanize community motivation toward increasing communal interactions [12]. This, in turn, could play a critical role in mitigating loneliness and its associated health risks, thereby contributing to the broader goal of promoting sustainable health conditions in rural contexts. Through this exploration, the study seeks to underscore the importance of community dialogue as a vital tool for enhancing rural populations' social and psychological resilience, paving the way for more vibrant and sustainable communities.

## Materials and methods

This cross-sectional study was performed with rural citizens who regularly visited a rural Japanese community hospital to clarify the association between the frequency of community dialogue and feelings of loneliness.

Setting

Situated in the southeastern Shimane Prefecture, Unnan City is a predominantly rural community within Japan. As of 2023, it was home to a population of 35,686, between 17,199 males and 18,487 females. A significant portion of this population, approximately 39%, comprised individuals aged 65 years and older, with projections suggesting that this demographic could expand to account for 50% by the year 2025. The healthcare infrastructure in Unnan City includes a variety of facilities, such as 16 clinics, a dozen home care centers, three stations for visiting nurses, and Unnan City Hospital. This hospital was equipped with 281 beds when the referenced study was conducted, broken down into 160 for acute care, 43 for comprehensive care, 30 for rehabilitation, and 48 for chronic care situations [13].

Within the city, care managers and home care workers bolstered the healthcare and support landscape. These professionals may operate independently or as part of the home care centers, focusing on coordinating care for patients in the home setting. They work closely with the patients, their families, and a network of healthcare and care professionals to tailor and oversee the delivery of care services. The duties of home care workers extend to providing physical care, assisting with daily living activities, and facilitating transportation, all aimed at enhancing the quality of life for those under their care [14].

Participants

Between September 1, 2023, and November 30, 2023, this study focused on individuals over 40 frequent visitors to the Department of General Medicine at Unnan City Hospital [15]. This research entailed extracting data from electronic medical records of patients who routinely sought care for chronic conditions or attended their annual health examinations at the hospital. Furthermore, to assess the levels of loneliness and engagement in community activities among these patients, they were provided with a survey based on the questionnaire of the Japanese version of the University of California, Los Angeles (UCLA) Loneliness Scale, which consists of three items designed to measure loneliness [16].

Data collection

Primary Outcome

The evaluation of loneliness levels among adults living in the community was carried out using the Japanese version of the UCLA Loneliness Scale, which comprised three questions. These questions are designed to measure feelings of companionship deficiency, exclusion, and isolation on a scale of 1-3 for each question, leading to a total score range of 3-9. The overall loneliness score was determined by adding the individual scores from these three questions [16].

Independent Variable

The investigation included an examination of community engagement through a specific survey question aimed at understanding the frequency of community interactions. Participants were asked about their regular engagement in conversations with friends and community members. The frequency of these dialogues was categorized into four levels: 'not at all or less than once a week' indicating the lowest level of dialogue, '2 to 3 times a week' indicating a low level of dialogue, '4 to 5 times a week' indicating a higher level of dialogue, and 'more than five times a week' signifying the highest level of community dialogue [15,17].

Covariate

Participants' background information was gathered from electronic health records at Unnan City Hospital [15]. This data collection focused on demographic and health-related parameters, including age, gender, body mass index (BMI) for nutritional status assessment, serum creatinine levels for evaluating renal function through estimated glomerular filtration rates (eGFR), and the Charlson Comorbidity Index (CCI). The CCI helps in gauging the severity of concurrent illnesses such as cardiovascular, respiratory, renal, liver diseases, diabetes, neurological conditions, autoimmune diseases, dementia, and cancer. These comprehensive data were sourced from the latest hospital visit records for chronic condition management or routine health screenings [18].

Statistical analysis

Parametric datasets were examined using Student's t-test, while the Mann-Whitney U test was employed to evaluate nonparametric datasets. The analysis further involved categorizing numerical variables into two groups based on median values. Specifically, the loneliness score was divided into higher loneliness (scores of 4 or above) and lower loneliness (scores below 4), grounded on the observation that the variable's mean and median were closely aligned (with an average score of 4.17, a standard deviation of 1.42, a median score of 4, and an interquartile range of 2). A univariate regression analysis was conducted to identify potential associations between community dialogue, various independent variables, and covariates. Following this, a multivariate logistic regression analysis was carried out to delve deeper into the relationship between the frequency of community dialogues and the propensity for higher levels of loneliness, incorporating only those variables that demonstrated a correlation with community participation in the univariate analysis (with a p-value threshold of less than 0.2 for inclusion). The analysis excluded participants for whom data were incomplete. A p-value of less than 0.05 was adopted to denote statistical significance. The statistical procedures were executed utilizing EZR, a user-friendly graphical interface for R, developed by Saitama Medical Center at Jichi Medical University in Saitama, Japan [19].

Ethical considerations

The hospital committed to maintaining the anonymity and confidentiality of all patient data in this research. Details pertinent to the study were made available on the hospital's website, ensuring no individual patient information was disclosed. Additionally, the website featured the contact information of a hospital representative to address any inquiries related to the study. All individuals participating in the study were adequately informed about its objectives and gave their informed consent before inclusion. The Clinical Ethics Committee of Unnan City Hospital approved the study's protocol, which was assigned the approval code: 20230010.

## Results

Participant selection

Between September 1, 2023, and November 31, 2023, 1,024 patients were regularly followed by the General Medicine Department [15]. The questionnaires were sent to all the patients. In total, 647 participants who answered the questionnaires were included in this study [15].

Participants’ demographic

The participants’ mean age was 71.26 years, with significant age variations across dialogue groups (p<0.001). Gender distribution remained relatively consistent, indicating no significant correlation between male sex percentage and dialogue frequency (p=0.222). Health indicators, including albumin levels and eGFR, showed no substantial differences across groups. Conversely, BMI demonstrated significant variations (p=0.001). Participants engaging in the most dialogue reported higher loneliness scores than those with the least dialogue (p<0.001). The CCI and the prevalence of chronic conditions such as heart failure, diabetes, and hypertension were analyzed, revealing no significant association with dialogue frequency, except for a noted variation in dyslipidemia prevalence, which was significantly associated with dialogue frequency (p=0.033) (Table 1).

**Table 1:** Demographics of the participants BMI, body mass index; Charlson Comorbidity Index, CCI; CKD, chronic kidney diseases; COPD, chronic obstructive pulmonary diseases; eGFR, estimated glomerular filtration rate; MI, myocardial infarction; SD, standard deviation

Factor	Total	Least dialogue	Less dialogue	More dialogue	Most dialogue	p-value
N	647	109	105	237	196	
Age, mean (SD)	71.26 (12.18)	67.80 (11.35)	72.48 (10.18)	74.18 (11.55)	68.98 (13.43)	<0.001
Male sex (%)	299 (46.3)	59 (54.1)	46 (43.8)	01 (42.6)	93 (47.7)	0.222
Albumin, mean (SD)	4.10 (0.41)	4.08 (0.55)	4.07 (0.38)	4.12 (0.36)	4.10 (0.39)	0.779
BMI, mean (SD)	23.00 (3.81)	4.31 (3.71)	22.85 (3.21)	2.54 (3.44)	22.89 (4.41)	0.001
eGFR, mean (SD)	63.93 (15.49)	66.30 (13.23)	63.45 (14.44)	62.92 (15.89)	64.08 (16.64)	0.299
Loneliness scale, mean (SD)	4.17 (1.42)	3.57 (0.98)	3.75 (1.12)	4.20 (1.34)	4.68 (1.67)	<0.001
Companionship, mean (SD)	1.54 (0.63)	1.27 (0.50)	1.33 (0.53)	1.56 (0.57)	1.78 (0.71)	<0.001
Isolated, mean (SD)	1.30 (0.51)	1.17 (0.40)	1.19 (0.44)	1.31 (0.48)	1.41 (0.60)	<0.001
Leftover, mean (SD)	1.33 (0.52)	1.14 (0.35)	1.23 (0.42)	1.33 (0.50)	1.49 (0.62)	<0.001
Higher loneliness (%)	337 (52.1)	34 (31.2)	41 (39.0)	133 (56.1)	129 (65.8)	<0.001
CCI ≥ 5 (%)	218 (33.7)	29 (26.6)	35 (33.3)	94 (39.7)	60 (30.6)	0.07
CCI (%)						
0	20 (3.1)	6 (5.5)	0 (0.0)	5 (2.1)	9 (4.6)	0.105
1	57 (8.8)	17 (15.6)	6 (5.7)	12 (5.1)	22 (11.2)	
2	82 (12.7)	13 (11.9)	13 (12.4)	26 (11.0)	30 (15.3)	
3	142 (21.9)	27 (24.8)	25 (23.8)	47 (19.8)	43 (21.9)	
4	128 (19.8)	17 (15.6)	26 (24.8)	53 (22.4)	32 (16.3)	
5	107 (16.5)	13 (11.9)	19 (18.1)	46 (19.4)	29 (14.8)	
6	64 (9.9)	10 (9.2)	12 (11.4)	27 (11.4)	15 (7.7)	
7	34 (5.3)	5 (4.6)	4 (3.8)	14 (5.9)	11 (5.6)	
8	10 (1.5)	1 (0.9)	0 (0.0)	5 (2.1)	4 (2.0)	
9	3 (0.5)	0 (0.0)	0 (0.0)	2 (0.8)	1 (0.5)	
Heart failure (%)	55 (8.5)	5 (4.6)	9 (8.6)	21 (8.9)	20 (10.2)	0.405
MI (%)	5 (0.8)	1 (0.9)	1 (1.0)	3 (1.3)	0 (0.0)	0.503
Asthma (%)	43 (6.6)	5 (4.6)	10 (9.5)	15 (6.3)	13 (6.6)	0.535
Peptic ulcer (%)	55 (8.5)	9 (8.3)	10 (9.5)	15 (6.3)	21 (10.7)	0.42
Kidney disease (%)	168 (26.0)	27 (24.8)	27 (25.7)	61 (25.7)	53 (27.0)	0.976
Liver disease (%)	52 (8.0)	9 (8.3)	10 (9.5)	22 (9.3)	11 (5.6)	0.498
COPD (%)	38 (5.9)	5 (4.6)	4 (3.8)	18 (7.6)	11 (5.6)	0.488
DM (%)	130 (20.1)	24 (22.2)	25 (23.8)	52 (21.9)	29 (14.8)	0.162
Brain infarction (%)	51 (7.9)	3 (2.8)	6 (5.7)	25 (10.5)	17 (8.7)	0.068
Brain hemorrhage (%)	13 (2.0)	4 (3.7)	0 (0.0)	5 (2.1)	4 (2.0)	0.297
Connective tissue disease (%)	85 (13.1)	15 (13.8)	12 (11.4)	31 (13.1)	27 (13.8)	0.945
dementia (%)	12 (1.9)	1 (0.9)	1 (1.0)	5 (2.1)	5 (2.6)	0.659
cancer (%)	69 (10.7)	10 (9.2)	11 (10.5)	27 (11.4)	21 (10.8)	0.942
Hypertension (%)	428 (66.2)	80 (73.4)	70 (66.7)	160 (67.5)	118 (60.2)	0.119
Dyslipidemia (%)	388 (60.0)	54 (49.5)	70 (66.7)	151 (63.7)	113 (57.7)	0.033

Multivariate logistic regression analysis

The multivariate logistic regression model underscored the significance of dialogue frequency on higher loneliness, with the odds of participating in community dialogue significantly increasing with dialogue frequency. Specifically, the ‘more frequency' and 'most frequency' groups were associated with higher loneliness compared to the 'least frequency' group (p<0.01). Furthermore, the model revealed a significant relationship between the increase in BMI and lower loneliness (p=0.023) and a reduced likelihood of higher loneliness among individuals with a CCI≥5 (p=0.031) (Table 2).

**Table 2:** The multivariate logistic regression model with higher loneliness and associated factors BMI, body mass index; Charlson Comorbidity Index, CCI; COPD, chronic obstructive pulmonary diseases; CI, confidential interval

Factor	Odds ratio	95%CI	P value
Community dialogue (reference: least frequency)			
Less frequency	1.29	0.73-2.29	0.39
More frequency	2.62	1.60-4.29	<0.01
Most frequency	4.11	2.47-6.85	<0.01
age≧75	1.2	0.81-1.77	0.36
BMI	0.95	0.91-0.99	0.023
CCI≧5	0.64	0.43-0.96	0.031
Diabetes	1.34	0.89-2.03	0.16
Dyslipidemia	0.86	0.61-1.21	0.38
Brain stroke	1.07	0.58-1.96	0.84

## Discussion

The study explored the dynamics of dialogue frequency among elderly patients and its relationship mainly with the degree of loneliness. By meticulously analyzing the responses of 647 participants, the research underscores the nuanced interactions between dialogue engagement and aspects such as loneliness, physical health, and the prevalence of chronic conditions. This discussion draws upon previous academic literature to deepen the understanding of these relationships and contextualize the findings within the broader geriatric social and health research field.

The significant variation in dialogue engagement with age, as demonstrated by the mean age differences across dialogue groups, resonates with the findings of previous articles, which noted the propensity for increased social interaction in rural adults as a coping mechanism for the social isolation commonly experienced in this demographic [20]. The lack of significant correlation between gender distribution and dialogue frequency aligns with previous research, suggesting that gender may not play a pivotal role in determining the quantity of social interaction among the elderly, contrary to earlier assumptions that women might engage in more social dialogue than men [21].

BMI and increased dialogue frequency, as well as its link to loneliness, extend the previous studies, which postulated that physical health status could significantly impact the social behavior of rural adults [22]. This relationship might reflect the social stigmatization surrounding obesity or the physical limitations it imposes, affecting the individual's social interaction and perceived loneliness [23]. However, this research shows that higher BMI is associated with lower loneliness. Many studies have been performed in Western cultures, so higher BMI can have a different and positive meaning in rural Japan [24]. The findings that higher BMI is significantly related to lower loneliness highlight the complex interplay between physical health, social engagement, and cultural issues.

Moreover, the study's observation that participants with more frequent dialogue reported higher loneliness scores corroborates the work of the previous studies, which emphasized the increases in frequent dialogue in communities, showing the degree of loneliness of people. This result can show different perspectives regarding cause-and-effect models [25]. When people feel lonely, they see others frequently [26]. Because of frequent meetings and comparing with others, people may feel lonely when realizing their differences [27]. This relationship underscores the importance of fostering the establishment of a diverse understanding of loneliness in community dialogue regarding social connections to mitigate feelings of loneliness and promote a sense of belonging in this vulnerable population [26,27]. An inappropriate increase in community dialogue can increase loneliness among some people in communities. When applying interventions, respect for the perception of people in communities is essential for appropriate interventions mitigating their loneliness.

The multivariate logistic regression model illuminated the intricate connections between dialogue frequency, loneliness, BMI, and comorbidity burden. The model demonstrated that increased dialogue frequency and lower BMI are associated with higher loneliness levels, whereas a higher CCI suggests a reduced likelihood of experiencing loneliness. These findings align with the hypothesis proposed by previous research that social and health-related factors interact in complex ways to influence the psychological well-being of the elderly, emphasizing the multifaceted nature of loneliness and the need for holistic approaches to addressing it [28]. As the previous article described, BMI can affect loneliness differently, depending on cultural and social issues [29]. In rural Japan, higher BMI can positively affect loneliness. High CCI can also have multiple relationships with various professionals [30]. Through interaction with them, people may reduce their loneliness. Thus, the degree of loneliness can be complicated by sociocultural factors and may cause different changes from other subjective health outcomes such as quality of life and self-rated health.

The primary limitation of this study lies in its cross-sectional design, which restricts the ability to establish causal relationships between dialogue frequency and the assessed outcomes, such as loneliness, companionship, and health indicators. The selection of participants from a single department of general medicine may also limit the generalizability of the findings to broader populations. Additionally, relying on self-reported measures for dialogue frequency and health outcomes could introduce bias. Future studies would benefit from longitudinal designs to clarify causation, broader participant recruitment for enhanced generalizability, and the inclusion of objective measures to validate self-reported data.

## Conclusions

This study highlights the significance of community dialogue in alleviating loneliness among rural Japanese populations, presenting compelling evidence that more frequent community interactions are inversely associated with feelings of loneliness. These findings suggest that initiatives to increase community dialogue need a nuanced approach to mental health and social cohesion in rural settings. The research further reveals an intriguing relationship between body mass index, the severity of comorbidities, and loneliness, offering insights into the complex interplay between physical health and social well-being. The importance of this study lies in its potential to inform policies and programs designed to foster social connections respecting rural contexts, thereby addressing the challenge of loneliness in rural communities.
